# Treatment Alternative and High Safety Profile of Acupuncture Plus Chemotherapy for Advanced Gastric Cancer

**DOI:** 10.1155/2022/8701779

**Published:** 2022-07-30

**Authors:** Xiaomei Miao, Hongying Wu, Yan Liu, Shu Zhang, Chaohui Li, Jie Hao

**Affiliations:** ^1^National Physician Hall, Cangzhou Central Hospital, Cangzhou, China; ^2^Department of Hepatopancreatobiliary Surgery, Cangzhou Central Hospital, Cangzhou, China; ^3^Department of Oncology, Cangzhou Central Hospital, Cangzhou, China

## Abstract

**Objective:**

The aim of this study is to evaluate the safety and tumor marker level changes of acupuncture plus chemotherapy (FOLFOX4) for advanced gastric cancer.

**Methods:**

One hundred and twenty patients with advanced gastric cancer who were treated at our hospital between May 2019 and April 2021 were recruited for prospective analysis, and all patients were allocated to the control and experimental groups in a 1 : 1 ratio using the random number table method, with 60 patients in each group. They received either chemotherapy using the FOLFOX4 regimen (control group) or the FOLFOX4 chemotherapy plus acupuncture (experimental group). Outcome measures included tumor marker levels, quality of life, and adverse events.

**Results:**

Before treatment, the two groups showed similar tumor markers levels and the MOS 36-item short-form health survey (SF-36) scores (*P* > 0.05). FOLFOX4 chemotherapy plus acupuncture was associated with significantly lower levels of carcinoembryonic antigen (CEA), carbohydrate antigen (CA) 19-9, and CA72-4 versus FOLFOX4 chemotherapy alone (*P* < 0.05). The patients who were given FOLFOX4 chemotherapy plus acupuncture showed significantly increased SF-36 scores versus monotherapy of the FOLFOX4 regimen (*P* < 0.05). The joint therapy resulted in a significantly lower incidence of adverse events versus the monotherapy (*P* < 0.05).

**Conclusion:**

Acupuncture plus chemotherapy using the FOLFOX4 regimen can effectively regulate the serum tumor marker levels of patients with advanced gastric cancer, with a high safety profile, which provides a viable treatment alternative.

## 1. Introduction

Gastric cancer is a malignant tumour disease with a very high incidence, ranking second in terms of incidence and mortality of malignant tumours in China and posing a serious threat to the health of the population [1–4]. Due to the atypical early symptoms of gastric cancer, the disease mostly progresses to advanced stages by the time of diagnosis [5, 6]. Chemotherapy is the main treatment modality for patients with inoperable advanced gastric cancer [7]. The FOLFOX4 regimen (oxaliplatin + 5-fluorouracil) is a common chemotherapy regimen used to treat patients with advanced gastric cancer and may also involve the use of calcium folinic acid injection (a sensitising agent for fluorouracil injection) [8]. Studies have shown that FOLFOX4 chemotherapy is safe and effective in reducing the pathological stage of gastric cancer, reducing postoperative recurrence and metastasis, and prolonging patient survival [9, 10]. Given the low selectivity in targeting tumour cells and the tendency to harm normal cells, chemotherapy may lead to adverse effects, reduced compliance, poor tolerance, and impaired therapeutic efficacy [11, 12].

Acupuncture provides disease management and health care by treating with acupuncture, whose stimulation of body acupoints regulates the function of breath power, blood, and internal organs [13, 14]. Acupuncture has been reported to reduce adverse events after chemotherapy in patients with gastric cancer, but few studies have combined it with the FOLFOX4 regimen. Therefore, the aim of this study was to evaluate the safety and changes in tumour marker levels of acupuncture combined with chemotherapy (FOLFOX4) in the treatment of advanced gastric cancer.

## 2. Materials and Methods

### 2.1. Baseline Data

One hundred and twenty patients with advanced gastric cancer who underwent consultation at our hospital between May 2019 and April 2021 were recruited for prospective analysis, and all patients were allocated to the control and experimental groups in a 1 : 1 ratio using the random number table method, with 60 patients in each group. All patients and their families were informed and asked to sign a consent form, and the study was approved for implementation by the ethics committee of Cangzhou Central Hospital, No. 297901-117.

### 2.2. Inclusion and Exclusion Criteria

#### 2.2.1. Inclusion Criteria

The inclusion criteria were as follows:

1. those who met the diagnostic criteria of gastric cancer in the Clinical Diagnostic and Treatment Guidelines Oncology Branch [15]
2. those who met the soft tissue tumor staging criteria for gastric cancer stage IIIB and stage IV by the American Joint Committee on Cancer (AJCC)
3. those with an expected survival of ≥3 months

#### 2.2.2. Exclusion Criteria

The exclusion criteria were as follows:

1. those with serious cardiovascular, cerebrovascular, hepatic, renal, and hematological diseases
2. those with cardiopulmonary dysfunction
3. those with brain metastases

### 2.3. Treatment Methods

The patients in the control group were given chemotherapy (FOLFOX4 regimen) [16]. Oxaliplatin injection (Qilu Pharmaceutical Co., Ltd.) 85 mg/m^2^ was given intravenously for 2 h on the first day, calcium folinate injection (Jiangsu Hengrui Pharmaceutical Co., Ltd.) 200 mg/m^2^ was given intravenously for 2 h on the first and second days, and 5-fluorouracil (Shanghai Xudong Haipu Pharmaceutical Co., Ltd.) 400 mg/m^2^ was pushed intravenously, followed by 2 h continuous intravenous of 5-fluorouracil 600 mg/m^2^. A course of treatment was given every 2 weeks for a total of 4 courses of treatment [17].

The patients in the experimental group were given acupuncture plus chemotherapy (FOLFOX4 regimen). A millineedle of 40 mm or more was used to perform acupuncture at the following acupoints, including Guanyuan, Qihai, Zusanli, Daheng, Neiguan, Xuehai, Diji, Shuidao, and Guilai, followed by the needling techniques of the lifting-thrusting method and the reinforcing-reducing method. A moxa stick about 2 cm long is placed above the needle handle, about 2-3 cm from the skin, lit from the lower end and the skin of the acupuncture point is covered with kraft paper, and the patient feels proper warmth at the point. The acupuncture takes about 20 minutes and the moxa strips are burnt out and the needles are withdrawn. During acupuncture, if the patient feels unbearable heat, a piece of cardboard can be placed over the acupuncture point to reduce the heat. Acupuncture was performed at Zusanli and Hangjian acupoints for patients with liver and stomach disharmony, at Zusanli, Piyu, Geyu, and Sanyinjiao for spleen and kidney yang deficiency. The needles remained at the points for 6 h. Acupuncture once every other day, 20 times as a course of treatment, a total of 28 courses of treatment.

### 2.4. Outcome Measures

1. Tumor markers: At the end of the procedure, 2–5 ml of fasting venous blood was collected from the patient, clotted, and centrifuged at 2500 r/min. The serum was separated to determine carcinoembryonic antigen (CEA), carbohydrate antigen (CA) 19-9, and CA72-4 using electrochemiluminescence immunoassay (ECLIA) with original matching reagents.
2. Quality of survival [18]: The MOS 36-item Short Form Health Survey (SF-36) was used to assess quality of life 3 months after the end of treatment in 8 domains: physical functioning, physical role, physical pain, general health, vitality, social functioning, role emotion, and mental health. The total score for each dimension is 100, with higher scores representing a better quality of life for the patient.
3. Adverse events: Adverse events, including anaemia, nausea and vomiting, malaise, leucopenia, and peripheral neuropathy, occurred during treatment in both groups were recorded and the incidence of adverse events was calculated.

### 2.5. Statistical Analysis

SPSS 22.0 was used for data analyses, and GraphPad Prism 8 was used for image rendering. The measurement data were expressed as ($\bar{x}$ ± *s*) and processed using the *t*-test. The count data were expressed as the number of cases (rate) and analyzed using the chi-square test. Differences were considered statistically significant at *P* < 0.05.

## 3. Results

### 3.1. Baseline Data

The baseline characteristics of the control group (33 males, 27 females, aged 38–63 (50.45 ± 5.68) years, 42 cases of stage IIIB, and 18 cases of stage IV) were comparable with those of the experimental group (31 males, 29 females, aged 39–66 (50.18 ± 5.94) years, 37 cases of clinical-stage IIIB, and 23 cases of stage IV) (*P* > 0.05) (Table 1).

### 3.2. Tumor Markers Levels

Before treatment, the two groups showed similar tumor markers levels (*P* > 0.05). FOLFOX4 chemotherapy plus acupuncture was associated with significantly lower levels of CEA, CA19-9, and CA72-4 (13.21 ± 1.31 *μ*g/ml, 158.14 ± 5.14 U/ml, 56.74 ± 5.27 U/ml) versus FOLFOX4 chemotherapy alone (16.25 ± 2.12 *μ*g/ml, 228.52 ± 9.14 U/ml, 70.08 ± 4.13 U/ml) (*P* < 0.001) (Table 2).

### 3.3. Quality of Life

**Figure 1:**
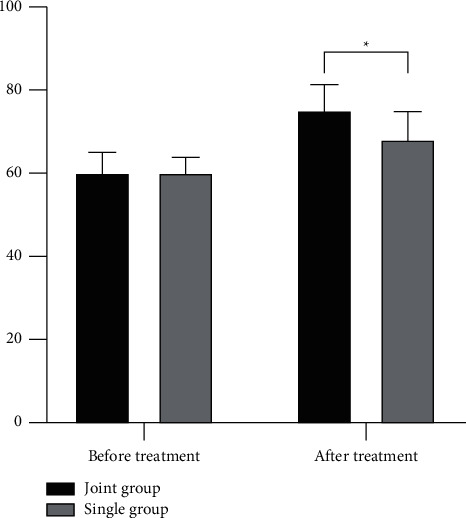
Comparison of SF-36 scores. ^*∗*^ indicates a statistically significant difference (*P* < 0.05) between the two groups.

The patients who were given FOLFOX4 chemotherapy plus acupuncture showed significantly increased SF-36 scores (75.23 ± 6.17) versus monotherapy of the chemotherapy (68.17 ± 6.88) (*P* < 0.001) (Figure 1).

### 3.4. Incidence of Adverse Events

The joint therapy resulted in a significantly lower incidence of adverse events (3.34%, including 1 (1.67%) case of nausea and vomiting and 1 (1.67%) case of fatigue) versus the monotherapy (41.67%, including 8 (13.34%) cases of anaemia, 11 (18.34%) cases of nausea and vomiting, 4 (6.67%) cases of fatigue, 1 (1.67%) case of leukopenia, and 1 (1.67%) case of peripheral neuropathy) (*P* < 0.05) (Table 3).

## 4. Discussion

Advanced gastric cancer refers to the invasion of tumour tissue into the stroma or stromal layer of the stomach or the occurrence of extrastromal metastasis. The main clinical manifestations are emaciation, epigastric pain, anaemia, loss of appetite, and corresponding clinical manifestations in distant sites. Currently, the FOLFOX4 regimen is a common chemotherapy regimen in clinical practice [19, 20]. Acupuncture features extensive indications and significant efficacy [21]. The results of this study showed a significant reduction in CEA, CA19-9 and CA72-4 levels after acupuncture combined with FOLFOX4 regimen chemotherapy, indicating the effectiveness of FOLFOX4 regimen chemotherapy in reducing tumour marker levels. It has been shown that CEA is highly sensitive to gastric cancer and its changes correlate with the sensitivity of gastric cancer to chemotherapy, showing great benefit in prognostic assessment and efficacy observation. CA19-9 is a highly specific gastrointestinal tumour-associated antigen whose expression correlates positively with the degree of tumour progression and has a sensitivity of approximately 40% for gastric cancer. CA72-4 is a tumour marker for gastrointestinal tract tumours and ovarian cancer and plays a role in detecting residual tumours and early gastric cancer recurrence. It has been confirmed in numerous studies that serum CEA, CA19-9, and CA72-4 are all common clinical serum tumor markers for gastric cancer, with considerable clinical significance in the diagnosis and treatment of gastric cancer. In traditional Chinese medicine, the process of disease development and regression is essentially the process of the struggle between the positive and evil breath power. Acupuncture plus chemotherapy has a wide range of indications and significant efficacy to better exploit its anticancer effects. Here, the patients given FOLFOX4 chemotherapy plus acupuncture showed significantly increased SF-36 scores and a lower incidence of adverse events versus monotherapy of the chemotherapy, indicating the benefits of quality of life after the intervention of acupuncture, as it stimulates the acupoints to unblock breath power and blood of the stomach, harmonize the Yin and Yang, and the dispel evil breath power, which resulted in enhanced quality of life and fewer adverse events.

The study of Xu demonstrated that acupuncture can reduce adverse events in gastric cancer patients after chemotherapy, for example, a large number of studies have shown that acupuncture at certain specific acupoints, such as the Neiguan point, not only has an effect on the release of wuqiang acid in the vomiting centre but also has a regulatory effect on the release of wuqiang acid in the local tissues of the gastrointestinal tract, providing a good antiemetic effect from effectively reducing damage to the gastric mucosa from chemotherapy drugs and restoring gastrointestinal motility [21]. It has also been shown that acupuncture can have a prestimulatory effect, i.e., some acupuncture can reduce the degree of damage when the body is injured only before the body is injured [22]. A related study by Zhou et al. also showed that acupuncture used early in chemotherapy was more effective in improving gastric motility than later in the course of chemotherapy, suggesting that if acupuncture is used early and at the right time in chemotherapy, it may prevent the onset of vomiting and nausea, or reduce its symptoms when it occurs [23].

However, there are obvious limitations to our study. First, our experimental sample was small, which may lead to some error in the results. Second, we need to improve the monitoring indicators in subsequent trials by adding indicators such as survival after treatment and the clinical remission rate of the efficacy evaluation criteria for solid tumours (RECIST) [24].

## 5. Conclusion

To sum up, acupuncture plus chemotherapy using the FOLFOX4 regimen can effectively regulate the serum tumor marker levels of patients with advanced gastric cancer, with a high safety profile, which provides a viable treatment alternative.

## Figures and Tables

**Table 1:** Comparison of baseline data ($\bar{x}$ ± *s*).

Groups	*n*	Gender		Age		Clinical stage	
		Male	Female	Range	Mean age	IIIB	IV
Control group	60	33	27	42–71	50.45 ± 5.68	42	18
Experimental group	60	31	29	40–72	50.18 ± 5.94	37	23
*t* value	—	0.251					
*P* value	—	0.802					

**Table 2:** Comparison of tumor markers levels ($\bar{x}$ ± *s*).

Groups	*n*	Before treatment			After treatment		
		CEA (*μ*g/ml)	CA19-9 (U/ml)	CA72-4 (U/ml)	CEA (*μ*g/ml)	CA19-9 (U/ml)	CA72-4 (U/ml)
Control group	60	24.23 ± 2.27	255.54 ± 8.17	78.25 ± 3.47	16.25 ± 2.12	228.52 ± 9.14	70.08 ± 4.13
Experimental group	60	24.51 ± 2.11	256.08 ± 7.94	77.94 ± 3.86	13.21 ± 1.31	158.14 ± 5.14	56.74 ± 5.27
*t* value	—	0.718	0.367	0.462	9.471	51.967	15.433
*P* value	—	0.474	0.714	0.645	<0.001	<0.001	<0.001

^*∗*^ indicates a statistically significant difference (*P* < 0.05) in the same group between before and after treatment.

**Table 3:** Comparison of incidence of adverse events (%).

Groups	*n*	Anemia	Nausea and vomiting	Fatigue	Leukopenia	Peripheral neuropathy	Total incidence
Control group	60	8 (13.34)	11 (18.34)	4 (6.67)	1 (1.67)	1 (1.67)	25 (41.67)
Experimental group	60	0 (0.00)	1 (1.67)	1 (1.67)	0 (0.00)	0 (0.00)	2 (3.34)
*χx* ^2^	—	25.281					
*P* value	—	<0.001					

## Data Availability

The datasets used during the present study are available from the corresponding author upon reasonable request.
